# Overweight and Obese Children's Ability to Report Energy Intake Using Digital Camera Food Records during a 2-Year Study

**DOI:** 10.1155/2012/247389

**Published:** 2012-08-16

**Authors:** Åsa Svensson, Maria Waling, Catharina Bäcklund, Christel Larsson

**Affiliations:** Department of Food and Nutrition, Umeå University, 901 87 Umeå, Sweden

## Abstract

The objective was to evaluate overweight and obese children's ability to report reproducible and valid estimates of energy intake (EI) by using digital camera food records (FR) during a 2-year study, compared with objectively measured total energy expenditure (TEE). Seventy-three overweight/obese children, aged 8–12 years at inclusion, kept FR with the help of digital cameras for 16 days in total, on 7 occasions during a 2-year period. On the same days, their TEE was registered with SenseWear Armband (SWA). The children underestimated their EI by −2.8 (2.4) MJ/d on the first assessment occasion (95% CI: −3.3, −2.3). Reporting accuracy did not differ between the 7 assessment occasions (*P* = 0.15). Variables negatively associated with reporting accuracy relative to TEE were increased age (95% CI: −0.07, −0.01) and BMI z-score (95% CI: −0.18, −0.06). Further, reporting accuracy relative to TEE was lower for girls than boys (95% CI: −0.14, −0.01) and on weekdays compared with weekend days (95% CI: −0.08, −0.001). In conclusion, overweight and obese children were able to report their EI using a digital camera FR with good reproducibility over a 2-year period, even though their EI was underestimated compared with objectively measured TEE.

## 1. Background

Estimated food record (FR) is one of the most common methods of dietary assessment. Its advantage is that food is recorded in real time and the method is thus not dependent on memory, as compared to the retrospective methods 24-hour recall and diet history interview [1]. The major disadvantages are that it is time consuming, strenuous, and can be inconvenient to conduct, although it is less so than the weighed FR. It has been suggested that children can reliably report their food intake from the age 8–10 years [2]. When children and adolescents use estimated FR to document their dietary intake, energy intake (EI) is usually underestimated [3]. Furthermore, it has been shown that overweight and obese children tend to underestimate their EI to a higher extent than their normal weight counterparts [2, 4]. More accurate and feasible methods are therefore needed when it comes to dietary intake assessment in children and adolescents in general and in overweight and obese children and adolescents in particular. The estimated FR could possibly be improved by using new technology [5], which is supposedly of interest for and easily adopted by young people. To photograph foods with a digital camera could be one way to make the recording of dietary intake easier and more attractive for children. Since children often eat away from home, for example, in school and at friends' places, the digital camera may facilitate documentation of what and how much is being consumed, so that it can more easily be recorded in a pen and paper FR later on. We are only aware of one study that used cameras to make it more feasible for children to report their food intake. The study showed that an FR based on photographs solely gave a similar estimate of EI to that from a traditional FR with pen and paper, but the participants found the photographic method quicker and easier to use [6]. However, a combination of the two methods may be benefited by the detailed pen and paper FR and the more convenient photographic method. 

When assessing dietary intake, the method used needs to be validated, not only in order to establish the trustworthiness of collected dietary data but also to determine if the method is suitable to be used in future studies in a similar population. Likewise, the reproducibility of the method used is of great interest since the method has to be reproducible in order to be valid. To evaluate the validity of a dietary assessment method, the assessed EI can be compared with the subject's total energy expenditure (TEE); if the subject is weight stable, EI should equal TEE [7]. The gold standard of measuring TEE is the doubly labeled water (DLW) method [3, 7]. This is, however, an expensive technique and other methods such as using accelerometers may be a more feasible alternative when having a large sample of subjects. The SenseWear Armband (SWA) (BodyMedia, Inc., Pittsburgh, PA, USA) is a multisensory device worn on the upper arm which consists of a two-axis accelerometer and sensors measuring skin temperature, near-body temperature, heat flux, and galvanic skin response, which together with computer software can be used to assess TEE. 

The objective of the present study was to evaluate overweight and obese children's ability to report reproducible and valid estimates of EI by using digital camera FR during a 2-year study, in comparison with TEE registered with SWA. 

## 2. Methods

### 2.1. Participants

Ninety-three overweight and obese children were recruited to a randomized controlled trial in Umeå, Sweden, from August 2006 to May 2007. Overweight and obesity was defined according to the International Obesity Task Force's age- and sex-specific BMI limits classifying a child as overweight at a BMI corresponding to an adult BMI of 25–29.9 kg/m^2^ and as obese corresponding to an adult BMI of ≥30 kg/m^2^ [8]. Details about the study, recruitment, and inclusion criteria have been described elsewhere [9]. In summary, the children, aged 8–12 years at inclusion, had been randomized to either an intervention or control group, and the same measurements were made in both groups regarding anthropometrics as well as dietary and physical activity assessments. Children in the intervention group also participated in 15 group sessions and used a web-based platform aimed at improving dietary habits and increasing physical activity. The study was approved by the Regional Ethical Review Board in Umeå.

### 2.2. Measurements

#### 2.2.1. Food Records

The children were asked to keep an estimated FR with the help of a digital camera on 7 occasions at regular intervals during a 2-year study period, altogether; six 2-day records and one 4-day record after one year of participation. The recording occasions were scheduled to cover weekdays and weekend days as well as different seasons throughout the years. On each occasion, the children were equipped with a digital camera, a measuring tape, a paper food diary, and a booklet “Matmallen” with pictures of common foods of different portion sizes and known weights [10]. The children were instructed to bring the camera and measuring tape with them during the day and to use it to photograph everything they ate and drank. They were instructed to place the measuring tape beside the food item or dish to help assessment of portion size when looking at the photographs at the end of the day. They were also instructed to take pictures of any retakes and leftovers. In the end of each record day, with the help of a parent, they were instructed to write down in the paper food diary everything that had been consumed during the day. As a memory aid and to help estimate portion sizes, they were instructed to look at the pictures in the digital camera. In the paper food diary, they noted time of meal, name of food (one per row), and any details of, for example, fat or sugar content, cooking methods, and brand label. They estimated portion sizes by comparing their own photographs to the ones in the picture booklet “Matmallen,” or by comparison with standard household measures and noted this in the diary. If they had not taken any pictures during the day, they had to rely on memory when completing the food diary. 

The children's EI was calculated by entering the food records into the nutrient analysis software Dietist XP version 3.1 (Kost och Näringsdata AB), which is based on the Swedish National Food Database and includes energy and nutrient content of over 2000 food items, dishes, and products. A trained dietician and two trained nutritionists entered the foods written in the diary and also checked the photographs for more details on type and amounts of food as this was not always clear from the paper food diaries. If the photographs did not show the same amounts of food that was written in the diary it was generally assumed that the diary was correct, that is, if the amount in the diary was not obviously wrong, it was assumed that the child did not finish the plate photographed. If there were photos of foods not mentioned in the diary, these were entered with the estimated amount visible in the photo. When no photographs were taken and amount of intake was not estimated of a food item written in the diary, standard portions given by the National Food Agency were used [11]. The dietician/nutritionists entering the data had agreed as far as possible on what type of food to enter when this was not clear from the record, for example, medium-fat milk was entered if the diary only said “milk” or if there was a picture of a glass of milk without any specific information given in the diary. Data from the food diaries were excluded from analysis if only one meal per day was recorded or if the child had a stomach illness during the day of recording, as these were considered incomplete days of recording and days unrepresentative of the child's habitual dietary intake, respectively.

#### 2.2.2. SenseWear Armband

The SWA Pro 2 and 3 (BodyMedia, Inc., Pittsburgh, PA, USA) was worn by the children on the same days as they conducted the FR. It was worn on the back of the upper right arm over the triceps muscle according to the manufacturer's recommendations and registered TEE at 1 minute intervals. The children were instructed to wear the SWA at all times 24 hours a day, except when water was involved, as in, for example, showering and swimming. During time off body, TEE was set equal to estimated basal metabolic rate, which was automatically estimated from the subject data (weight, height, sex, and age) entered in the software. The computer software InnerView Professional version 5.1 (BodyMedia, Inc., Pittsburgh, PA, USA) was used to estimate TEE from the armbands' registrations together with information about the child's age, sex, weight, and height. To be included in analysis, the SWA had to be worn for at least 19 hours (≥80%) of the day. This cutoff was chosen arbitrarily and the wearing time for records of TEE that did not meet this criterion was in the range of 0–79% of the day. 

#### 2.2.3. Anthropometric Measurements

Weight and height were measured by a research nurse using standardized procedures during a visit to Umeå University Hospital at inclusion and after one and two years of participation. Weight and height were measured with the child wearing light clothing. BMI (kg/m²) was calculated and BMI *z*-scores were computed based on an American child reference population [12], a Swedish child reference population [13], and a mixed child reference population defined by the WHO [14]. Weight status was determined according to the standards of the International Obesity Task Force [8].

### 2.3. Data Analysis and Statistics

Twenty children either did not hand in any records, handed in records that did not meet the inclusion criteria, or handed in records of either food intake or TEE only, thus not enabling a comparison of EI and TEE. In total, 73 children and 288 records of EI and TEE were included, and the number of included assessment days was 583. The first assessment occasion was regarded as the reference category; therefore, assessments of 7 children that had their first assessment on the second possible occasion were moved, so that the first assessment occasion included all 73 children. Any following assessment occasions for those 7 children were likewise moved so that the time between the occasions remained unchanged. Twelve children (16.5%) participated on all seven assessment occasions, 8 (11%) on six occasions, 16 (22%) on five occasions, 4 (5.5%) on four occasions, 8 (11%) on three occasions, 11 (15%) on two occasions, and 14 (19%) on one occasion.

Statistical analysis was performed in IBM SPSS Statistics version 19 and *P* values ≤0.05 were considered significant. In the text, results are presented as mean (SD). Differences in characteristics at inclusion between girls and boys, children in intervention and control group, and overweight and obese children were analyzed using the independent samples *t*-test (age), Mann-Whitney *U* test (BMI *z*-score), and chi-square test (sex, study group, and weight status). Differences in proportions of girls and boys, children in intervention and control group, as well as body weight status at inclusion compared with after one and two years of participation, respectively, were analyzed using the chi-square test. The Spearman correlation coefficient was calculated for EI and TEE since TEE was not normally distributed. Difference between EI and TEE at the first assessment occasion was analyzed using the one sample *t*-test, and a Bland-Altman plot was used to display the agreement of estimated EI with TEE. The reproducibility of the method when used on several occasions was analyzed using mixed model analysis, to take into account the missing values of EI-TEE on the different assessment occasions. EI-TEE was the dependent variable, and since the Bland-Altman plot showed a dependence of the reporting accuracy on the average energy values, an additional model was performed with the dependent variable (EI-TEE)/TEE. In both models, assessment occasion and a dichotomous variable indicating whether or not a weekend day was present in the record were repeated variables. Study group, age, sex, and BMI *z*-score at inclusion calculated from reference values [14] were included to investigate their influence on reporting accuracy. All independent variables were treated as fixed effects and a Bonferroni correction was applied to adjust for the repeated comparisons. The covariance structure compound symmetry was used since it fitted the data best. 

## 3. Results

The children's characteristics measured at inclusion and after one and two years of participation are shown in Table 1. There was no difference in age between the sexes at inclusion. The boys, however, had higher BMI *z*-scores than the girls at inclusion (*P* ≤ 0.05), and the obese children were older than the overweight children (*P* = 0.02). Two children became normal weight between recruitment and measurement at inclusion. After one year of participation, the proportion of normal weight children had increased from 2.7 to 17.5% (*P* = 0.004), and this difference remained after two years of participation. There was no statistically significant difference in any of the characteristics between children in control and intervention groups and there was an approximately even distribution of children in respective groups at inclusion as well as after one and two years of participation (data not shown). 

The children recorded on average 17 (5) foods per day and photographed 11 (6), or 65%, of these foods. On average, the children gave estimated amounts of intake in the paper food diary for 13 (6), or 74%, of the recorded foods per day. On 29 out of 288 assessment occasions (10%), the children did not photograph, although they noted foods in the diary, and on 4 occasions (1%) they did not note any foods in the diary although they photographed foods. On 55 (19%) of the 288 assessment occasions, 80% or more of the foods were photographed and given estimated amounts of intake in the paper food diary. On 210 (73%) of the assessment occasions at least 50% of the foods were photographed and given estimated amounts in the diary. 

**Figure 1:**
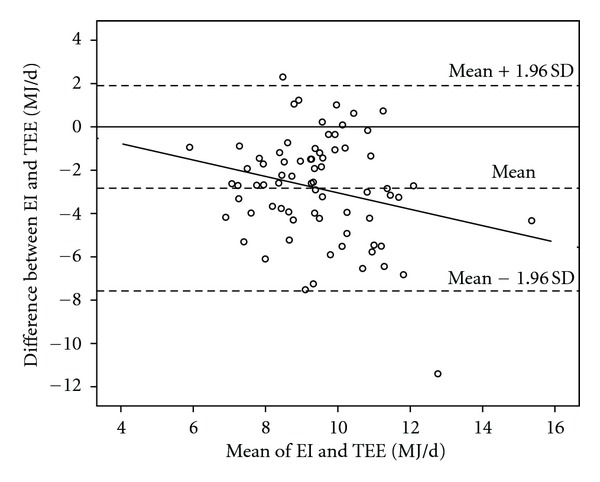
Difference between assessed energy intake (EI) from estimated food records aided by digital camera and total energy expenditure (TEE) registered with Sense Wear Armband in 73 children on the first assessment occasion, against the mean of the two variables. The correlation coefficient of EI-TEE and mean of EI and TEE was −0.24 (*P* = 0.04) and the regression equation was *y* = 0.74 ± 0.38 *x* (*P* = 0.04).

On the first occasion, which was used to investigate the children's ability to accurately estimate their EI, the children underestimated their EI compared to TEE with −2.8 (2.4) MJ/d (*t *(72) = −10.0, CI: −3.4, −2.3), or 24% (Table 2). Five children (7%) estimated their EI within +5% of the individually measured TEE, 6 children (8%) overestimated their EI, and 62 children (85%) underestimated their EI.  Figure 1 shows the difference between EI and TEE on the first assessment occasion in a Bland-Altman plot. The limits of agreement were −7.6 to 1.9 MJ/d and assessments of two children were outside these limits. 

Table 2 shows the estimated EI and registered TEE on the 7 occasions as well as the difference between EI and TEE. The difference ranged from −3.9 MJ/d (32%) on the third assessment occasion to −2.7 MJ/d (23%) on the second assessment occasion and the correlation coefficient for EI and TEE was only statistically significant on assessment occasion 4 (*ρ* = 0.34, CI: 0.04, 0.58), although it was borderline significant on the first assessment occasion. Mixed model analysis showed no significant differences in reporting accuracy between the 7 assessment occasions (*F* = 1.60, *P* = 0.15) (Table 3). When reporting accuracy was the dependent variable, there were significant negative associations with increased age (*b* = −0.61, CI: −0.98, −0.25) and BMI *z*-score at inclusion (*b* = −1.89, CI: −2.67, −1.11). Furthermore, when reporting accuracy relative to TEE was the dependent variable, there were also significant associations with sex and the presence of a weekend day in the record, with girls reporting with a lower accuracy than boys (*b* = 0.07, CI: −0.14, −0.01) and with lower accuracy on weekdays than weekend days (*b* = 0.04, CI: −0.08, −0.001). There was no difference in reporting accuracy between intervention and control group (Table 3).

## 4. Discussion

The children in the present study were able to estimate their EI with high reproducibility over a 2-year period, even though EI was underestimated, by 24% on the first assessment occasion. Younger children as well as children with a lower BMI and TEE were more able to estimate their EI accurately. Furthermore, the boys estimated their EI more accurately than the girls, and underestimation was lower when a weekend day was included in the record.

Systematic reviews on dietary assessment in children and adolescents have concluded that individuals who are overweight and obese tend to underestimate their EI more than normal weight subjects [2, 4]. Nevertheless, the 24% underestimation of EI found in the present study using digital cameras is lower than the ones shown in previous validation studies of traditional (not using digital cameras) estimated FR in overweight and obese adolescents [15–17]. Bandini et al. showed a 41% underestimation of EI from a 2-week traditional estimated FR compared with TEE measured with DLW in obese adolescents aged 12–18 years [15]. However, in that study the long recording period most certainly contributed to a high underreporting as the motivation to record food intake is likely to decline over time. In a more recent study, Singh et al. showed a 35% underestimation of EI when using a traditional estimated FR during 9 days compared with TEE measured with DLW in 12–15-year-old overweight adolescents [17]. In the two above-mentioned studies, the participants were somewhat older than in the present study, and it has been observed that underestimation of EI has a tendency to increase with age [2, 18]. A validation result similar to that of the present study was shown by Champagne et al. who validated a traditional estimated 8-day FR in 10-year-old children against TEE measured with DLW and found that the obese children underestimated their EI with 25% [16]. 

Validation studies of traditional estimated FR in normal weight children and adolescents have shown an underestimation of EI of 12, 19, and 21% when compared with TEE [15, 16, 19]. Validation studies of EI among children and adolescents recruited from the general population (including both normal weight, overweight, and obese) against TEE measured with DLW have shown both underreporting and over reporting of EI; Swedish 15-year-old adolescents underestimated their EI with 20% when using a traditional estimated FR [20], and parents of Australian 6–9-year-old children overestimated their children's EI with 4% when using a traditional estimated FR [21]. In a study by Higgins et al., underreporting of EI was 16% when comparing a 3-day traditional estimated FR with known weights of the foods eaten in 10–16-year-old children and adolescents [6]. In the same study, the children also used digital cameras to record their food intake and this method gave similar results as the traditional estimated FR. 

Underestimation of EI could result from, for example, a conscious unwillingness to bother with the recording or admit what is eaten and/or that the participants simply forget to record some foods. This in turn could result either from methodological factors (e.g., feasibility or that the children were tired from participating in many different activities and measurements in the study) or individual factors (e.g., social desirability). Another source of error is the difficulty to accurately estimate portion size. Since underreporting can have many different causes, it is not likely that the problem will be solved entirely by making the dietary assessment method more user friendly; however, doing so perhaps could lessen the problem. The method used in the present study aimed at being more feasible for children than traditional FR and resulted in a more accurate estimate of EI; however, the possible attained improvement was not great enough to contribute to a valid estimation of EI. The children in the present study were overweight or obese, and about half of them took part in an intervention program with focus on improving dietary habits and physical activity. It would be understandable if the children wanted to report the desired dietary habits, especially the children in the intervention group. The children in the intervention group could also have been in a negative energy balance as a result of participating in the intervention program. However, results of the intervention study after one year showed no difference in BMI between intervention and control group [22], and accuracy of estimated EI did not differ in the intervention group compared to the control group. The positive energy balance due to growth in all children could have masked some of the underestimation of EI in the present study. However, the amount of extra energy needed for growth during the 2 respective 4 days that the assessment of EI and TEE was conducted was probably negligible. Furthermore, all children in the present study took part in an extensive study protocol with additional measurements to the ones described in this paper, which put more effort on them to complete the repeated FR.

Previous studies have shown that a higher BMI may be associated with lower accuracy of estimated EI among overweight and obese children [3, 9]. Also age has been shown to be associated with reporting accuracy of EI in children and adolescents [2, 18]. These results were confirmed by the present study. A possible reason for the association between higher TEE and higher underestimation of EI is that the more a person needs to eat to maintain energy balance, the more effort it takes to report everything eaten. Since older and heavier subjects by consequence have higher energy needs, age and BMI could seem to be more important factors behind underreporting than they really are if TEE is not adjusted for. The result of the present study indicates that TEE explains some, but not all, of the underreporting in older and heavier children. This is also in line with results from a study on normal weight preadolescent girls in which TEE was controlled for, and where TEE and age were found to be independently positively associated with underreporting of EI [19]. 

Although it has more often been shown that underestimation of EI does not differ between the sexes [15, 20, 21], the present study showed that girls underreported more than boys. However, this was only seen after TEE was controlled for since the boys had higher TEE than the girls. Also in a previous study where EI was assessed with traditional estimated FR, girls were shown to underreport more than boys [16]. 

In the present study, the children used digital cameras as an aid to record their food intake, and 65% of the recorded items were photographed. The children were instructed to photograph everything they ate or drank but some of them did not feel comfortable with bringing the digital camera to school or to friends' places and therefore left it at home. As a consequence, some children focused the recording on writing in the paper food diary in the evening. This could have made it more difficult for them to estimate the portion size. On the other hand, some children seemed to have enjoyed taking pictures, and these children used the digital camera a lot, but they did not always note everything they photographed in the paper food diary. On four assessment occasions, none of the foods photographed were noted in the diary, but it was more common that a few photographed foods per assessment occasion were not noted or given estimated amounts of intake. Even though most of the children noted what had been eaten in the diary, it was fairly common that they did not estimate amounts for some foods. For those items (on average 24% of recorded items), a trained dietician/nutritionist estimated the amount of food in the picture. In this study three different persons made the estimates and entered the dietary data into the nutrient analysis software and this could have introduced some bias as they did probably not interpret foods and amounts in the pictures in the same way, even though standardized procedures were aimed for. For those food items in the diary where no estimates of amounts were made and no photographs were taken, the trained dietician/nutritionists used standard portions given by the National Food Agency [11]. Errors in estimating portion size could bias the results in both directions, and it is not known who best could estimate amounts, the child who ate the food, the parent, or the educated dietician/nutritionist.

Even though children aged 8–12 years are able to record their food intake on their own [2], it seems likely that parents' involvement is important to encourage the children to actually complete the FR. The fact that the children in this study estimated their EI more accurately on weekends compared to weekdays could be due to the fact that families with school aged children often are less busy on weekends than weekdays. On weekdays many families participate in scheduled leisure time activities in addition to school and work, which leaves less time and energy to sit down in the evening and complete the FR. Also, on weekends parents have better knowledge of the child's food intake than on weekdays when meals are eaten in school, which makes it easier for them to help with the recording. 

In the present study, TEE was measured using SWA, which has been validated against DLW in Swedish overweight and obese children recruited from the same study as the present [23]. The SWA showed to be valid on a group level, but the results differed on the individual level.

A limitation of the present study is the few days of assessment per occasion. On 6 of the 7 occasions, subjects were instructed to record their EI and measure TEE for only two days. A person who is in energy balance does not necessarily have an intake that equals expenditure each day; rather, this varies over time. Studies show that EI in children may have a high day-to-day variability [24], and therefore habitual EI and TEE are best estimated from several days of measurement. With few days of assessment, however, one avoids the decline in motivation which occurs when several assessment days are used [25].

## 5. Conclusions

Overweight and obese children were able to report their EI using digital camera FR with good reproducibility over a 2-year period, even though EI was underestimated by 24% compared with objectively measured TEE. The ability to report EI with higher accuracy was found in the younger, the less overweight, and the boys. Furthermore, reporting accuracy was higher on weekends versus weekdays and decreased with higher TEE. Future research ought to focus on developing ways of using new technology, for example, mobile phones, to make it more attractive and feasible for children and adolescents to validly report their food intake using FR.

## Figures and Tables

**Table 1:** Characteristics of children at inclusion and after one and two years of participation, respectively, presented as mean (SD) or percentage proportion.

	Inclusion (*n* = 73)	Year 1 (*n* = 57)	Year 2 (*n* = 54)
Age, y	10.4 (1.0)	11.4 (1.0)	12.4 (1.0)
Weight, kg	50.6 (10.0)	55.8 (11.4)	61.7 (11.7)
Height, cm	148.1 (8.4)	154.2 (8.9)	160.3 (9.1)
BMI, kg/m²	22.8 (2.6)	23.2 (2.9)	23.8 (2.9)
BMI *z*-score, CDC^1^	1.9 (0.8)	1.6 (0.9)	1.4 (0.5)
BMI *z*-score, Sweden^2^	3.0 (1.3)	2.6 (1.3)	2.4 (1.2)
BMI *z*-score, WHO^3^	2.0 (0.5)	1.8 (0.6)	1.7 (0.7)
Proportion obese, (%)^4^	27.4	17.5	16.7
Proportion overweight, (%)^4^	69.9	65.0	64.8
Proportion normal weight, (%)^4^	2.7	17.5	18.5
Proportion girls, (%)	46.6	49.1	44.4

^1^Calculated in comparison with an American child reference population [12].

^2^Calculated in comparison with a Swedish child reference population [13].

^3^Calculated in comparison with a mixed child reference population [14].

^4^Weight status according to the International Obesity Task Force [8].

**Table 2:** Assessed energy intake (EI) from estimated food records aided by digital camera and total energy expenditure (TEE) registered with SenseWear Armband, of children on 7 occasions during 2 years. Data are presented as mean (SD), [95% CI], correlation coefficient, or percentage proportion, and the difference between EI and TEE was analyzed using the one sample *t*-test and Spearman's correlation.

Assessment occasion	1	2	3	4	5	6	7	Mean of all 288 records
	(*n* = 73)	(*n* = 46)	(*n* = 41)	(*n* = 42)	(*n* = 31)	(*n* = 28)	(*n* = 27)	
EI, MJ/d	8.03 (1.72)	7.89 (1.53)	7.62 (1.95)	7.37 (1.59)	7.93 (1.68)	8.31 (1.59)	7.66 (1.78)	7.83 (1.70)
TEE, MJ/d	10.87 (2.19)	10.61 (2.03)	11.49 (1.98)	10.42 (1.87)	10.68 (2.51)	11.53 (2.52)	10.67 (2.66)	10.87 (2.22)
EI/TEE	0.76 (0.19)	0.77 (0.19)	0.68 (0.19)	0.72 (0.15)	0.77 (0.19)	0.75 (0.23)	0.76 (0.27)	0.74 (0.20)
EI-TEE, MJ/d	−2.84 (2.42)	−2.72 (2.38)	−3.87 (2.47)	−3.05 (2.01)	−2.75 (2.54)	−3.22 (3.09)	−3.01 (3.30)	−3.04 (2.55)
	[−3.40, −2.27]	[−3.42, −2.01]	[−4.65, −3.09]	[−3.68, −2.42]	[−3.68, −1.81]	[−4.41, −2.02]	[−4.32, −1.70]	
Correlation coefficient of EI and TEE	0.23	0.19	0.16	0.34	0.35	−0.02	−0.16	0.17
	[0.00, 0.44]	[−0.11, 0.46]	[−0.16, 0.45]	[0.04, 0.58]	[−0.004,0.63]	[−0.39, 0.36]	[−0.51, 0.23]	
EI-TEE_Weekdays,_ MJ/d	−3.00 (2.71)	−2.83 (2.68)	−3.95 (2.66)	−3.33 (2.45)	−3.44 (3.06)	−3.69 (3.57)	−3.77 (3.72)	−3.35 (2.90)
EI-TEE_Weekends,_ MJ/d	−2.71 (3.23)	−1.75 (3.15)	−2.84 (3.73)	−2.74 (3.28)	−1.18 (2.43)	−1.72 (3.90)	−0.64 (2.10)	−2.23 (3.19)
Proportion weekends in records, (%)	43	18	21	43	43	26	20	33

**Table 3:** Mixed model analyses of possible differences in accuracy of assessed energy intake (EI) from estimated food records aided by digital camera compared with total energy expenditure (TEE) registered with SenseWear Armband, of children on 7 occasions during 2 years. Analyses were conducted with regard to presence of a weekend day in the record, group belonging (intervention or control group), sex, age, and BMI *z*-score.

	Model with dependent variable EI-TEE	Model with dependent variable (EI-TEE)/TEE
	*b* (95% CI)	*b* (95% CI)
Assessment occasion 1 (*n* = 73)	Reference	Reference
Assessment occasion 2 (*n* = 46)	0.09 (−1.06, 1.24)	0.002 (−0.09, 0.10)
Assessment occasion 3 (*n* = 41)	−1.04 (−2.23, 0.16)	−0.09 (−0.18, 0.01)
Assessment occasion 4 (*n* = 42)	−0.19 (−1.37, 1.00)	−0.04 (−0.13, 0.06)
Assessment occasion 5 (*n* = 31)	0.04 (−1.29, 1.36)	0.00 (−0.11, 0.11)
Assessment occasion 6 (*n* = 28)	−0.33 (−1.73, 1.06)	−0.003 (−0.12, 0.11)
Assessment occasion 7 (*n* = 27)	−0.22 (−1.61, 1.18)	−0.001 (−0.12, 0.11)
Weekend day in record	0.42 (−0.07, 0.90)	0.04 (0.001, 0.08)^∗^
Group^1^	0.06 (−0.70, 0.82)	0.02 (−0.04, 0.08)
Sex^2^	−0.50 (−1.32, 0.32)	−0.07 (−0.14, −0.01)^∗^
Age	−0.61 (−0.98, −0.25)^∗∗∗^	−0.04 (−0.07, −0.01)^∗∗^
BMI *z*-score^3^	−1.89 (−2.67, −1.11)^∗∗∗^	−0.12 (−0.18, −0.06)^∗∗∗^

^1^Reference category: control group.

^2^Reference category: boys.

^3^Calculated in comparison with a mixed child reference population [14].

^∗^Significance at *P* ≤ 0.05.

^∗∗^Significance at *P* ≤ 0.01.

^∗∗∗^Significance at *P* ≤ 0.001.

## References

[1] Gibson RS (2005). *Principles of Nutritional Assessment*.

[2] Livingstone MBE, Robson PJ, Wallace JMW (2004). Issues in dietary intake assessment of children and adolescents. *British Journal of Nutrition*.

[3] Burrows TL, Martin RJ, Collins CE (2010). A systematic review of the validity of dietary assessment methods in
children when compared with the method of doubly labeled water. *Journal of the American Dietetic Association*.

[4] Forrestal SG (2011). Energy intake misreporting among children and adolescents: a literature review. *Maternal and Child Nutrition*.

[5] Ngo J, Engelen A, Molag M, Roesle J, García-Segovia P, Serra-Majem L (2009). A review of the use of information and communication technologies for dietary assessment. *British Journal of Nutrition*.

[6] Higgins JA, LaSalle AL, Zhaoxing P (2009). Validation of photographic food records in children: are pictures really worth a thousand words?. *European Journal of Clinical Nutrition*.

[7] Schoeller DA (2002). Validation of habitual energy intake. *Public Health Nutrition*.

[8] Cole TJ, Bellizzi MC, Flegal KM, Dietz WH (2000). Establishing a standard definition for child overweight and obesity worldwide: international survey. *British Medical Journal*.

[9] Waling MU, Larsson CL (2009). Energy intake of swedish overweight and obese children is underestimated using a diet history interview. *Journal of Nutrition*.

[10] Håglin L, Hagman U, Nilsson M (1995). Evaluation of the meal model ’Matmallen’. A means of estimating consumed amounts of food. *Scandinavian Journal of Nutrition*.

[11] Livsmedelsverket (1999). *Vikttabell*.

[12] Kuczmarski RJ, Ogden CL, Grummer-Strawn LM (2000). CDC growth charts: United States. *Advance Data*.

[13] He Q, Albertsson-Wikland K, Karlberg J (2000). Population-based body mass index reference values from Göteborg, Sweden: birth to 18 years of age. *Acta Paediatrica*.

[14] De Onis M, Onyango AW, Borghi E, Siyam A, Nishida C, Siekmann J (2007). Development of a WHO growth reference for school-aged children and adolescents. *Bulletin of the World Health Organization*.

[15] Bandini LG, Schoeller DA, Cyr HN, Dietz WH (1990). Validity of reported energy intake in obese and nonobese adolescents. *American Journal of Clinical Nutrition*.

[16] Champagne CM, Baker NB, DeLany JP, Harsha DW, Bray GA (1998). Assessment of energy intake underreporting by doubly labeled water and observations on reported nutrient intakes in children. *Journal of the American Dietetic Association*.

[17] Singh R, Martin BR, Hickey Y (2009). Comparison of self-reported and measured metabolizable energy intake with total energy expenditure in overweight teens. *American Journal of Clinical Nutrition*.

[18] Forrestal SG (2011). Energy intake misreporting among children and adolescents: a literature review. *Maternal and Child Nutrition*.

[19] Bandini LG, Cyr H, Must A, Dietz WH (1997). Validity of reported energy intake in preadolescent girls. *American Journal of Clinical Nutrition*.

[20] Bratteby LE, Sandhagen B, Fan H, Enghardt H, Samuelson G (1998). Total energy expenditure and physical activity as assessed by the doubly labeled water method in Swedish adolescents in whom energy intake was underestimated by 7-d diet records. *American Journal of Clinical Nutrition*.

[21] O’Connor J, Ball EJ, Steinbeck KS (2001). Comparison of total energy expenditure and energy intake in children aged 6–9 y. *American Journal of Clinical Nutrition*.

[22] Waling M, Lind T, Hernell O, Larsson C (2010). A one-year intervention has modest effects on energy and macronutrient intakes of overweight and obese Swedish children. *Journal of Nutrition*.

[23] Bäcklund C, Sundelin G, Larsson C (2010). Validity of armband measuring energy expenditure in overweight and obese children. *Medicine and Science in Sports and Exercise*.

[24] Nielsen SB, Montgomery C, Kelly LA, Jackson DM, Reilly JJ (2008). Energy intake variability in free-living young children. *Archives of Disease in Childhood*.

[25] Moreno LA, Kersting M, De Henauw S (2005). How to measure dietary intake and food habits in adolescence: the European perspective. *International Journal of Obesity*.

